# Automated Design Methodology for Electromagnetic Energy Harvesters

**DOI:** 10.3390/s25113358

**Published:** 2025-05-27

**Authors:** Bianca Leistritz, Ludwig Herzog

**Affiliations:** IMMS Institut für Mikroelektronik- und Mechatronik-Systeme Gemeinnützige GmbH, 98693 Ilmenau, Germany; ludwig.herzog@imms.de

**Keywords:** electromagnetic energy harvester, design automation, comparison of structures

## Abstract

An automated design methodology for electromagnetic harvesters is necessary for the cost-effective design of adapted energy harvesters to be used in application-specific requirements. With the help of the automated design methodology presented in this paper, application-specific designs can be generated quickly and easily, and systematic comparisons between the structures can be made to select the best suited harvester structure. For the dimensioning of the design parameters, both analytical approximation formulas and models for coupling with field calculations were implemented. An exemplary structure comparison based on three scenarios shows the advantageous suitability of one topology in large areas of the aspect ratio.

## 1. Introduction and State of the Art

Due to the increasing monitoring and optimization of processes, more and more sensor and actuator systems are being used in a wide variety of applications. For greater flexibility, these are often supplied as energy self-sufficient wireless systems using batteries. The use of energy harvesters can significantly extend the service life of batteries or even enable battery-free operation [1]. In addition to energy transmission via RFID, kinetic energy harvesters offer promising possibilities, particularly in industrial environments and in logistics, where mechanical movements are omnipresent. For required energies in the range of a few hundred microwatts to a few milliwatts, the mechanical–electrical energy conversion of electromagnetic systems is often discussed, especially for relatively low-frequency excitations [2].

For several years, research activities have been concerned with supplying wireless sensor systems with the aid of energy harvesters. In addition to piezoelectric and capacitive converters, electromagnetic energy harvesters, which convert mechanical energy into electrical energy through a relative movement between a magnetic field and a coil, are very promising. However, in order to use electromagnetic energy harvesters efficiently, the related design must be adapted to the respective application. In previous studies, there are overviews of different prototypes of electromagnetic harvesters [3]. For the realization of new products, there is no systematic approach to facilitate the selection of an optimal basic structure according to the specific application requirements. So far, only comparative studies that consider specific boundary conditions with regard to excitation, installation space and aspect ratio have been presented [4,5,6].

In [7], different structures without back iron have already been examined for a design space of 7.7 cm^3^, which corresponds to an AA battery, and for a design space of 4 cm^3^. The excitations were selected in a low-frequency range of about 10 to 30 Hz, which is typical for many industrial applications. Analytical formulas exist for these ironless structures, assuming the simplification, which is that $\mu_{r}=1$ applies in the entire range. The agreement between analytical models and FEM results is quite good. An analytical description is more complicated in the case of ferrous structures. For them, no analytical description of the magnetic flux density anywhere in space is possible, except for an approximation of the flux density in the gap using a network model [8]. Magnetic modeling with FEM tools, such as the commercial variants COMSOL and Ansys Maxwell, or free tools, such as FEMM, is therefore typical.

In the current work, an automated design tool is presented. It enables both the script-based design for a specific application and the topology comparison for various application scenarios regarding maximum output power. The structure comparison as well as the automated design enable a reduction in the development time and thus also of the necessary costs. The aim is to open up new application scenarios for electromagnetic energy harvesters for wider use in industrial and everyday applications.

The implementation was carried out in python version 3.10 with a connection to Ansys Maxwell 2022 R1 using the python library pyANSYS 0.7.5. The direct link between analytical and FEM calculations enables a continuous design process. A study was then carried out using the automated design methodology implemented. Various topologies were compared with each other using different scenarios in terms of design space and excitation, with the goal of increasing the power density of the harvester.

## 2. Overall System

**Figure 1:**
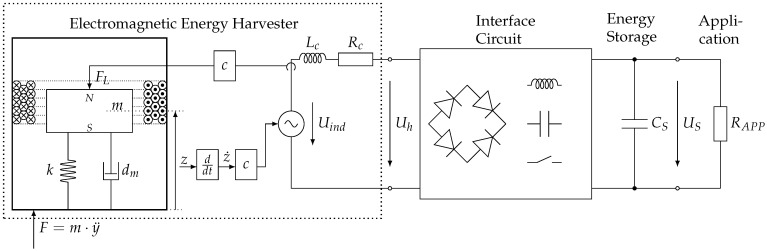
System overview of an electromagnetic harvester.

The electromagnetic energy harvesters considered in this paper are kinetic energy converters. Kinetic energy harvesters are usually connected to a vibrating component. In most cases, a sinusoidal excitation is assumed. As a result of the inertia, a moving mass within the harvester is deflected, which is specifically electrically damped, and thus, mechanical energy is converted into electrical energy. In Figure 1, a system overview is shown.

In the case of electromagnetic converters, this takes place by means of electrodynamic voltage induction when the magnetic field enclosed by a coil changes. In the simplest case, an electromagnetic harvester consists of a magnet and a coil. More complex topologies with several components and additional magnetic flux conductors, as shown in Section 5, are also possible.

In order to supply an energy self-sufficient system with energy, the mechanically–electrically converted energy usually has to be rectified and temporarily stored so that power peaks can be provided, for example, when a sensor system is sending data. For this purpose, the energy conversion from the mechanical domain to the electrical domain must be supplemented by an electronic interface circuit that takes into account and fulfills the operating point requirements of the sensor system. These interface circuits must ensure power-optimized operation of the energy converter and efficient storage of the extracted ambient energy in a storage element such as a double-layer capacitor, a fuel cell or a rechargeable battery. An advanced energy management system monitors the available energy over time and adjusts the operation of the system accordingly. Ref. [9] provides an overview of load matching techniques and integrated circuits for maximum power point tracking.

The focus of the current work is on the electromagnetic converter. An optimal ohmic load resistance is considered for the calculation of the output power [10].

## 3. Methods and Model Description

The design of electromagnetic energy harvesters is based on the analytical description of the physical relationships of the mechanical–electrical transducer system. As a result of external excitation $F=m\;\cdot \;\ddot{y}$, the moving assembly with a mass *m* is deflected. As shown in Figure 1, this is usually the magnetic circuit with the permanent magnets and the back iron. The deflection of the moving mass can be described as a spring-mass-damper system. The spring constant is defined by *k*. The damping of an energy harvester generally consists of a mechanical damping $d_{m}$ and an electrically induced damping. Mechanical damping refers to energy loss due to material friction in spring or guide elements, or also due to air friction. In electromagnetic energy harvesters, the electrical damping is caused by the Lorentz force $F_{L}$. This is generated by a current flowing through the coil when the coil is loaded. According to Lenz’s rule, the force opposes the deflection of the mass *m* and inhibits the oscillation. The equilibrium of forces results in the differential equation for the internal deflection of the mass *z*:

(1) $$m\ddot{z}+d_{m}\dot{z}+kz+F_{L}=-m\ddot{y}$$

In inertia-excited systems, the housing experiences an excitation that is assumed to be sinusoidal in the first step. The literature mostly reports on resonant energy converters whose natural frequency corresponds directly to the excitation frequency. Although purely sinusoidal excitation is rarely encountered in practical applications, this simplified approach allows initial insights into the performance parameters. For a purely sinusoidal excitation with

(2) $$y\left(t\right)=\hat{Y}sin\left(\omega t\right)$$

the equation of motion (1) gives the following general solution in the time domain:

(3) $$z\left(t\right)=\hat{Z}sin\left(\omega t+\phi \right)$$

The deflection of the moving magnetic circuit leads to a time-varying magnetic field in the coil. Based on Faraday’s law of induction, the relative movement between an electrical conductor and a magnetic field causes the generation of an induction voltage, which is directed in the opposite direction to its cause. Depending on the flux linkage Ψ, respectively, the multiplication of the number of windings *N* and the magnetic flux Φ, the induced voltage $U_{ind}$ is obtained from

(4) $$U_{ind}=-\frac{d\Psi }{dt}=-N\frac{d\Phi }{dt}$$

The temporal change of the magnetic flux can be expressed by the product of a geometry-dependent local change of the magnetic flux and the application-specific speed:

(5) $$U_{ind}=-N\frac{d\Phi }{dz}\dot{z}$$

The proportionality factor between the induction voltage and the speed is usually referred to as the coupling factor *c*, which can be applied as follows:

(6) $$c\left(z\right)=N\frac{d\Phi }{dz}$$

The magnetic flux results from the integral of the magnetic flux density $\vec{B}$ by an area $\vec{A}$:

(7) $$\Phi =\int \vec{B}\;\cdot \;d\vec{A}$$

This shows that a change in flux is caused by a change in area or a change in magnetic flux density. In most implementations, the effective area of the coil is constant, and the location-dependent magnetic flux density is used.

However, it is not the absolute value of the magnetic flux that is decisive for calculating the induced voltage, but rather, the local change in the magnetic flux. Since magnetic field lines are closed due to the source-free nature of the B field,

(8) $$\oint \vec{B}\;d\vec{A}=0,$$

the difference between two magnetic fluxes through the base and cover of a cylinder can be calculated using the radial flux through the lateral surface. This results in the following for the averaged magnetic flux in a coil with the height $h_{coil}$:

(9) $$\frac{d\Phi_{z,m}\left(z\right)}{dz}=-\frac{1}{h_{coil}}\Phi_{r}\left(z\right)$$

with

(10) $$\Phi_{r}\left(z\right)=\int_{z-\frac{h}{2}}^{z+\frac{h}{2}}2\pi R\;\cdot \;B_{r}\left(\bar{z}\right)\;d\bar{z}$$

The integral over the coil height can be described by the mean value of the radial flux density:

(11) $$\frac{d\Phi_{z,m}\left(z\right)}{dz}=-2\pi R\;\cdot \;B_{r,m}\left(z\right)$$

with

(12) $$B_{r,m}\left(z\right)=\frac{1}{h}\int_{z-\frac{h}{2}}^{z+\frac{h}{2}}B_{r}\left(\bar{z}\right)\;d\bar{z}$$

The average magnetic flux density over the coil is determined by the specific geometric dimensions of the magnetic circuit geometry, which are usually calculated using a coupled FEM.

When an interface circuit or an ohmic resistor is connected to the harvester, a current flows through the coil, which in turn causes a Lorentz force that is directed in the opposite direction to the cause and dampens the deflection:

(13) $$\vec{F_{L}}=I\left(\vec{l}\times \vec{B}\right)$$

The axial magnitude of the Lorentz force can be calculated using the radial magnetic flux density and can thus also be described using the coupling factor (Equation (6)):

(14) $$F_{L}\left(z\right)=I\;\cdot \;l\;\cdot \;B_{r,m}\left(z\right)=i\;\cdot \;N\;\cdot \;2\pi R\;\cdot \;B_{r,m}\left(z\right)=-i\;\cdot \;N\;\cdot \;\frac{d\Phi_{z,m}\left(z\right)}{dz}=-i\;\cdot \;c\left(z\right)$$

The current flowing is dependent on the interface circuit, which attempts to extract the maximum power from the system using maximum power point tracking. The maximum power can be calculated by assuming an optimum ohmic resistance as a load. In this case, the overall system can be written as a system of differential equations:

(15) $$m\ddot{z}+d_{m}\dot{z}+kz-c\left(z\right)i=-m\ddot{y}$$

(16) $$\left(R_{c}+R_{load}\right)\;\cdot \;i=-c\left(z\right)\;\cdot \;\dot{z}$$

If Equation (16) is converted to *i* and then inserted into Equation (15), it becomes clear that the Lorentz force causes a speed-dependent damping similar to mechanical damping, where the damping value is not constant but dependent on the deflection:

(17) $$m\ddot{z}+d_{m}\dot{z}+kz-\frac{c{\left(z\right)}^{2}}{R_{c}+R_{load}}\dot{z}=-m\ddot{y}$$

The maximum power that can be extracted from an optimum ohmic resistor is as follows:

(18) $$P_{max}=R_{load}\;\cdot \;i^{2}=\frac{R_{load}}{{\left(R_{c}+R_{load}\right)}^{2}}{\left(c\left(z\right)\;\cdot \;\dot{z}\right)}^{2}$$

## 4. Realization of the Automated Design

The individual parameters that influence the output power cannot be selected independently of each other. The aim when designing energy harvesters is therefore to calculate the dimensions of magnets, coils and back iron in such a way that everything is arranged in the specified installation space, taking into account the necessary deflection, and thus, maximum output power is achieved. However, this cannot be written down using a simple analytical equation but must be determined using optimization methods.

For this purpose, an automatic design tool has been developed using python. In addition to the analytical functions, the mean radial magnetic flux in the coil according to Equation (12) is required to determine the coupling factor *c*. For this purpose, a coupling to ANSYS Maxwell is realized via pyAEDT [11] for the magnetic field simulation.

**Figure 2:**
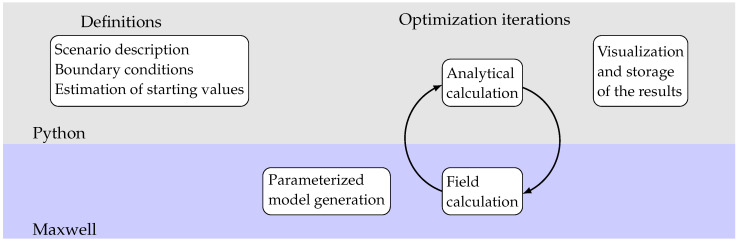
Scheme of coupling.

The general procedure is shown schematically in Figure 2. First, all boundary conditions are defined. In addition to the description of the application with design space and excitation, these also include material specifications and minimum values for geometric dimensions as well as, for example, the minimum output voltage as a requirement of the electronics. Table 1 shows an overview of the necessary boundary conditions and values used. The minimum dimensions and the intended gap between the coil and magnet depend primarily on the manufacturing process. The smaller the gap can be, the more efficient the harvester becomes, at the expense of higher demands on the production, assembly and transverse stiffness of springs. The mechanical damping in the system has an important influence on the system behavior. In order to be able to compare the current study with values from the literature, the value of 0.1 kg/s was selected, as in the [4]. The minimum voltage depends on the interface circuit. Lower voltages tend to enable more efficient designs, but can lead to power losses in the electronics. An optimum overall efficiency must therefore be the goal.

**Figure 3:**
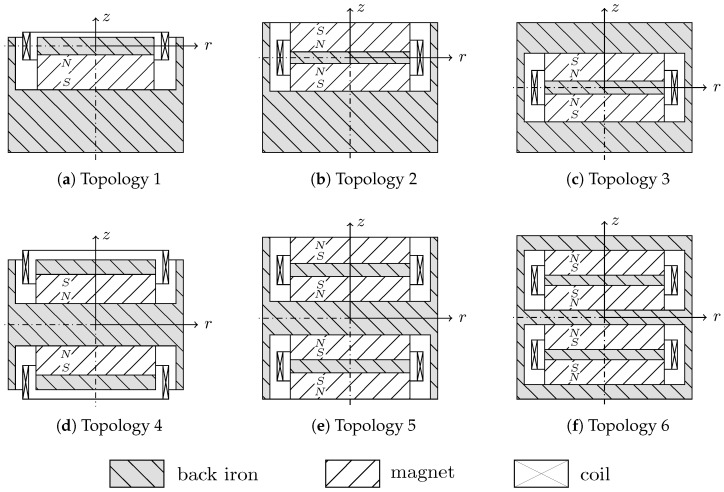
Topologies.

The topology to be considered must also be selected for the automatic design process. The various topologies differ in the number and arrangement of magnets and coils. The topologies currently under investigation are shown in Figure 3. These are standard topologies for a cylindrical design space and with increasing complexity, especially with regard to production.

Topologies 1 to 3 are the basic topologies. In topology 1, only one magnet is combined with a steel disk as a magnetic flux conductor. The coil is arranged in the center of the disc in the equilibrium position. As a result, the flux change is different in both directions of movement. Topology 2 is an extension with an opposing magnet, and topology 3, with the enclosing return, is a fully symmetrical arrangement. Topologies 4 to 6 are symmetrical extensions of the first three variants. The basic topological structures are implemented as parameterized functions. The scope is modularly expandable.

**Figure 4:**
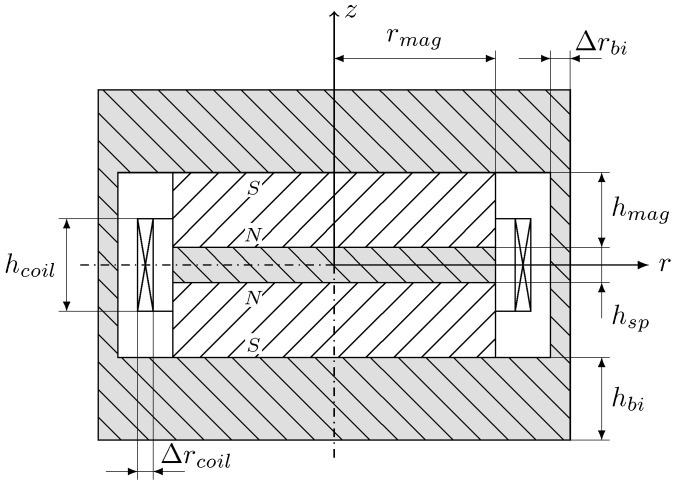
Example parameter definition for topology 3.

In order to design a simulation model to be optimized, a set of parameters has to be defined, which fully describes the harvester topology. The presented topologies can be described using just a limited number of parameters, which ensures simple application. These parameters are shown as an example in Figure 4 for topology 3. The geometric dimensions for the magnets ($r_{mag}$ and $h_{mag}$), the spacer ($h_{sp}$) and the coils ($\Delta r_{coil}$ and $h_{coil}$) are the free optimization variables. The back iron dimensions ($\Delta r_{bi}$ and $h_{bi}$) are variables dependent on the outer dimensions of the harvester.

Depending on the selected topology and the design space specifications, initial values are estimated for the geometry parameters to be optimized. For this purpose, empirical values for the ratios between each other are used from previously performed optimization calculations. For topology 3, for example, the magnet height ($h_{mag}$) is initialized with 20%, the height of the spacer ($h_{sp}$) with 10%, and the back iron height ($h_{bi}$) with 25% of the total installation height.

**Figure 5:**
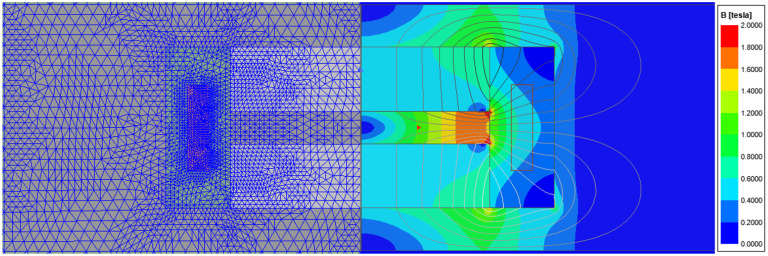
Example of meshing, magnetic flux density and flux lines for topology 3.

An initial parameterized Maxwell model is created based on the initial values determined. The geometry variables serve as design variables so that they can be varied as part of a parameter study. The required materials are defined and the structure is generated according to the topology. In addition, a boundary is generated and the meshing and setup properties are defined. Furthermore, the average flux density within the coil is created using a field calculator. In order to generate sufficiently accurate results with as few iterations of Maxwell’s automatic mesher as possible, a fine initial mesh was defined for the coil in particular. Figure 5 shows the exemplary mesh, the magnitude of the magnetic flux density and the flux lines for the demonstrator presented in Section 6, which is based on topology 3.

As part of the optimization iterations, the analytical calculation of the output power was carried out in python 3.10 and, based on this, the optimization function provided new geometry variables. These were used to overwrite the design variables in Maxwell 2022 R1. Once the Maxwell model was analyzed again, the flux density was read out. This resulted in a cycle until the termination criterion was reached. At the end, the result was visualized and all determined variables were saved in a file.

## 5. Exemplary Comparison

**Figure 6:**
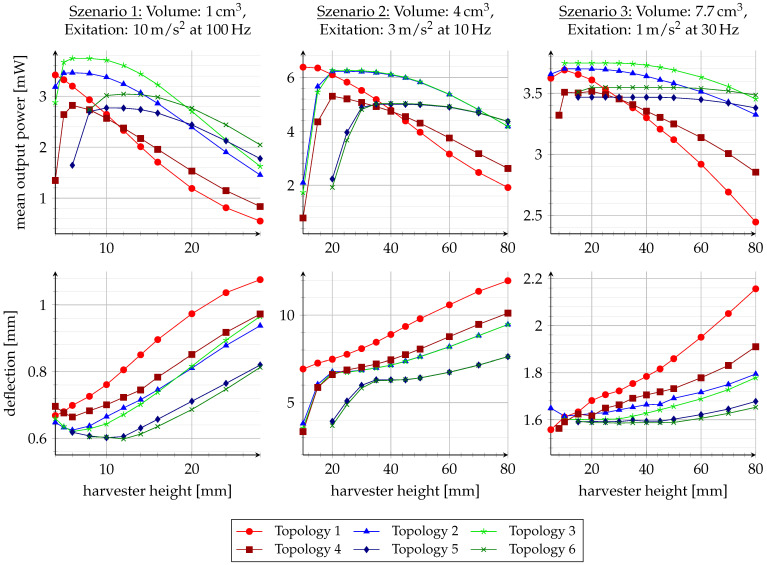
Results of the comparison study.

The implemented design automation is used here to exemplarily compare the different topologies for three different application scenarios. The application scenarios are chosen for comparison with existing studies [4,7] with a volume of 1 cm^3^, an excitation amplitude of 10 m/s^2^ and excitation frequency of 100 Hz as the first scenario; 4 cm^3^, 3 m/s^2^ and 10 Hz as the second scenario; and 7.7 cm^3^, 1 m/s^2^ and 30 Hz as the third scenario, respectively. The influence of the aspect ratio is also investigated for the different volumes. For this purpose, the installation space height is varied and the diameter is adjusted accordingly. Finally, the geometric parameters are optimized for each simulation point. The results are shown in Figure 6. In addition to the dependence of the mean output power on the height of the harvester, the deflection of the moving mass is also shown for all scenarios.

The diagrams show that each topology has an optimal aspect ratio depending on the scenario. If the structure is too low or too high, the power density decreases. This is, for instance, influenced by the minimum distances between the coil and magnet. A reduction in the coupling factor is evident not only from a lower average output power, but also from a greater deflection due to the lower damping.

In contrast to previous investigations on ironless structures, no significant difference is found in the presented study between the suitability of topologies for scenarios with relatively small deflections compared to large deflections. In all scenarios, topology 3 achieves the highest output power over a large range of the harvester’s height. For very flat structures, topology 1 is recommended. In most cases, however, the electrical damping of topology 1 is quite low, which leads to a large internal deflection and a low output power. Topology 2 usually delivers only slightly less output power than topology 3. Both structures achieve optimum performance over a larger range of the harvester height.

It is to be expected that the symmetrical structures would deliver better output power for tall, elongated harvesters. However, the intersections in the power curves only occur at quite high harvester heights, which generally leads to poorer output power due to their very small footprint. In specific application scenarios, the evaluation of the topologies can take into account not only the output power but also aspects such as the reduction in stray fields and manufacturing costs.

## 6. Measurements

A demonstrator was produced to validate the calculations carried out. As the demonstrator will also be used by the research partner to test front-end circuits, there are requirements for the power range, eigenfrequency and the minimum voltage available. The selected output power range of a few milliwatts corresponds to typical values for the supply of radio modules for IoT applications. According to the comparison of the different structures, topology 3 was chosen for the demonstrator.

The demonstrator was manufactured in-house, which ensures a cost-effective and well-controlled outcome. For manufacturing reasons, the minimum air gap and the minimum width of the coil were increased to 1 mm and the used wire diameter to 80 µm. The remaining boundary conditions were retained according to Table 1. The required transducer volume is approximately 10 cm^3^. The calculated properties of the demonstrator are summarized in Table 2 and the resulting geometric dimensions in Table 3.

The results show, among other things, that the width of the coil corresponds to the minimum width. In the study carried out, this was often observed when only the maximum output power was optimized. Therefore, if necessary, it is important to specify a minimum voltage. Furthermore, in the case of the demonstrator, it can also be seen that the optimum results produce thick dimensions for the back iron. This is due to the fact that more magnetic materials can no longer significantly increase the magnetic flux above a certain size. However, as the density of the steel is somewhat greater than that of the magnetic material in the materials under consideration, this results in dimensions that generate a larger mass. This increases the deflection and, therefore, also the speed. In the case of the demonstrator, this is more efficient in order to increase the coupling factor.

**Figure 7:**
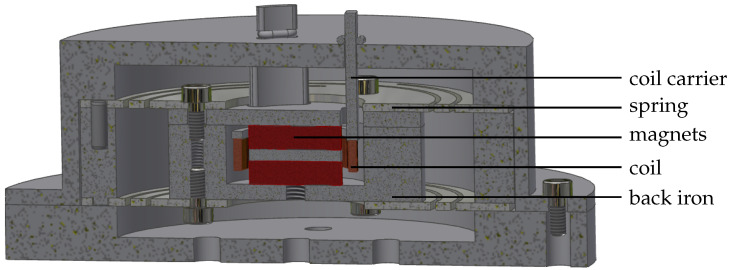
Demonstrator design.

In addition to the electromagnetic transducer itself, a suspension, a coil holder and a housing are required. The overall design of the demonstrator is shown in Figure 7.

Commercially available magnets are used for the electromagnetic transducer system. The back iron is designed in two parts, whereby, as usual with topology 3, one part must have openings for the coil holder to pass through. The coil holder itself is designed as a 3D-printed part. It is attached to the housing using three mounting elements. The housing is also 3D-printed. The suspension is implemented using a structured flat spring, which is dimensioned with regard to stiffness and tension at maximum deflection. It is manufactured by sheet metal laser cutting.

**Figure 8:**
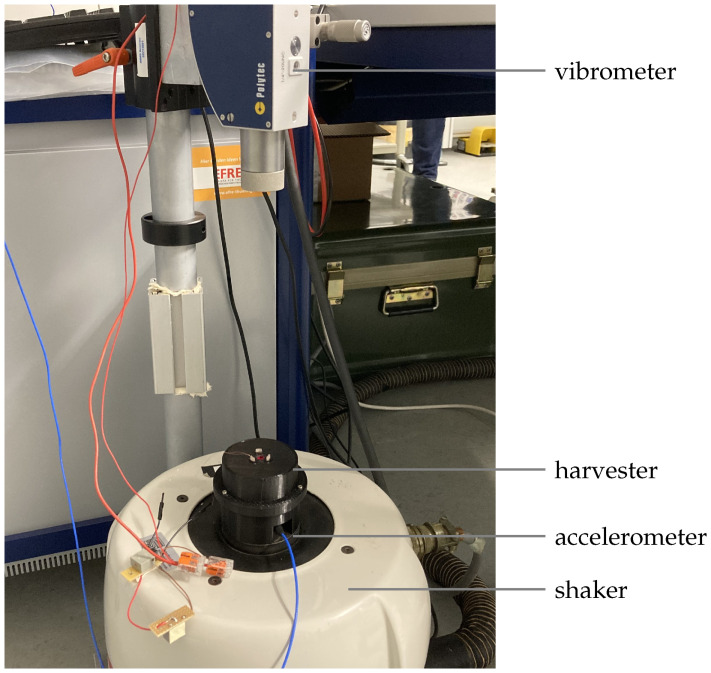
Measuring setup.

The demonstrator was measured on a measuring stand consisting of a shaker, an acceleration sensor and a vibrometer (see Figure 8). A controller system was used to control the acceleration in a closed loop. The housing was provided with an opening for the direct measurement of the internal deflection of the actual wall system. This allowed the vibration of the back iron to be recorded with the vibrometer.

The measurement results show agreement with the modeled values and significant deviations. The natural frequency of the demonstrator was determined to be 99 Hz, which corresponds sufficiently to the desired value as described above. But the output voltage achieved and the associated output power were very low. More detailed investigations revealed that the vibration amplitude of the moving mass vie is too low, and therefore, the mechanical damping of the structure is significantly higher than the values assumed in the literature. In order to investigate this behavior further, the purely mechanical damping was determined with the vibrometer and the harvester in idle mode (no electrical damping). Depending on the excitation amplitude and the resulting different deflections of the internal mass, the damping value was determined between d= 1 and 2 kg/s. This results in a range for Lehr’s damping factor of 0.01 to 0.02. It was also observed that the damping increases with increasing deflection. However, if the higher damping in the system is taken into account and the optimum load resistor is adjusted accordingly, the measurement results for the output voltage across the load resistor and the calculated power match the simulation values very well.

**Figure 9:**
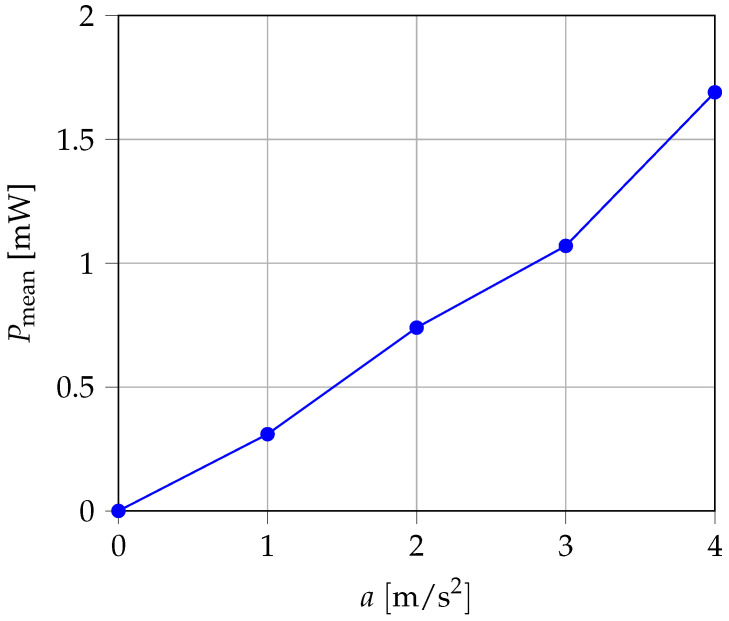
Dependency of output power from acceleration.

The output power determined for the various excitation amplitudes is shown in Figure 9. With constant mechanical damping, the converted power should increase quadratically with the acceleration amplitude. However, due to the increasing damping with greater vibration amplitude, an almost linear curve resulted instead.

**Figure 10:**
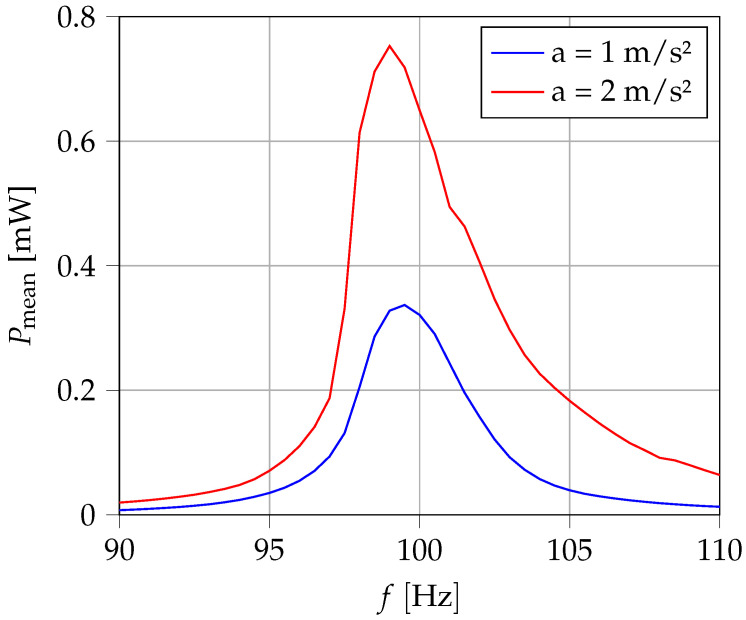
Frequency dependency of output power.

In Figure 10, the dependence on the excitation frequency was also measured for two different acceleration amplitudes. A noticeable asymmetry can be seen in the resonance range. The output power decreases significantly more at lower frequencies than at higher frequencies. The bandwidth is approx. 4 to 5 Hz. The causes may be non-linear spring behavior or frequency-dependent or amplitude-dependent damping behavior.

Overall, the demonstrator was able to show that the actual electromagnetic transducer can be precisely calculated using the automatic design tool that was implemented. Taking into account the higher and amplitude-dependent damping, the average output powers match quite well, which means that an exact modeling of the coupling factor can be concluded. However, there is still a need for optimization in the prediction of the mechanical damping of the suspension, which was not part of this work.

## 7. Conclusions

By implementing a python program library, an automated design process for electromagnetic energy harvesters in combination with a spatial magnetic field simulation software was realized. This design flow can be extended modularly by further topologies at any time. This enabled a coupling between analytical calculations and the calculation of the magnetic flux density by means of field simulations.

The exemplary investigations carried out on three application scenarios, which differ in particular in their deflections, show the general applicability of the approach. A starting expectation was that this approach could lead to optimal topologies based on the defined application scenario. A core finding of this study is that, however, there is no essential dependence of the topology to be selected on the application requirements. In the calculations carried out, topology 3 always achieved the highest output power for all investigated application scenarios.

In addition to a structural comparison, the automated design methodology also serves to design the harvesters quickly and therefore cost-effectively, which is a core demand for industry applications. In order to lower implementation effort and to make it easier to begin with this methodology, it should be examined whether the field calculations can be carried out with an open source FEM tool, such as FEMM, as no very complicated field calculations are necessary. This would further reduce the costs for dimensioning the harvesters and possibly open up new areas of application.

## Figures and Tables

**Table 1:** Boundary conditions.

Description	Value	Unit
Minimum dimensions	0.5	mm
Wire diameter	40	µm
Copper fill factor	0.6	1
Specific electrical resistance	17.05	mΩ·mm^2^/m
Gap between coil/magnet	0.5	mm
Coercive field strength	−875 300	A/m
Remanence flux density	1.1	T
Density of magnet	7.6	g/cm^3^
Material of back iron	steel_1010	
Density of steel	7.85	g/cm^3^
Mechanical damping	0.1	kg/s
Minimum voltage	0.8	V

**Table 2:** Properties of the demonstrator.

Description	Value	Unit
Excitation frequency *f*	100	Hz
Excitation amplitude *a*	0.7	m/s^2^
Inner displacement $z_{amp}$	0.4	mm
Moving mass *m*	68.7	g
Coil resistance $R_{coil}$	76	Ω
Optimal load resistance $R_{load}$	920	Ω
Output voltage $V_{RMS}$	1.6	V
Output power $P_{mean}$	2.6	mW

**Table 3:** Geometric dimensions of the demonstrator.

Description	Value	Unit
Magnet radius $r_{mag}$	6	mm
Magnet height $h_{mag}$	3	mm
Spacer height $h_{sp}$	1.5	mm
Coil width $\Delta r_{coil}$	1	mm
Coil height $h_{coil}$	4	mm
back iron width $\Delta r_{bi}$	7.5	mm
back iron height $h_{bi}$	2	mm

## Data Availability

The data that support the findings of this study are available from the corresponding author, upon reasonable request.
